# Paraneoplastic Cerebellar Degeneration from Subclinical Breast Cancer on Mammography

**DOI:** 10.31662/jmaj.2025-0234

**Published:** 2025-09-26

**Authors:** Shinsuke Sasada, Ayumi Kawamata

**Affiliations:** 1 Department of Surgical Oncology, Research Institute for Radiation Biology and Medicine, Hiroshima University, Hiroshima, Japan

**Keywords:** breast cancer, paraneoplastic cerebellar degeneration, anti-Yo antibody, SPECT, dedicated breast PET

Paraneoplastic cerebellar degeneration (PCD) is a rare presentation of tumor immune-mediated cerebellar ataxias ^(1), (2), (3)^. PCD often occurs with breast and gynecologic cancer (anti-Yo and anti-Ri antibodies), small cell lung cancer (anti-Hu and anti-CV2/ collapsin response-mediator protein 5 [CRMP5]), and Hodgkin’s lymphoma (anti-Tr antibodies) ^(4)^. A 45-year-old female with PCD developed worsening gait impairment and increased cerebellar perfusion on brain single photon emission computed tomography (SPECT) (Figure 1A). The anti-Yo antibody was present in the cerebrospinal fluid. Left breast cancer was subclinical on mammography (Figure 1B); however, it was detected via dedicated breast positron emission tomography (dbPET) (Figure 1C). She underwent a mastectomy and was diagnosed with invasive ductal carcinoma with an invasive diameter of 3 mm. The intensity of cerebellar hypermetabolism decreased on brain SPECT a week post-surgery (Figure 1D). She remained symptomatic without further progression. Patients with PCD symptoms and specific antibodies require appropriate diagnostic evaluation to identify the underlying cause.

**Figure 1.**
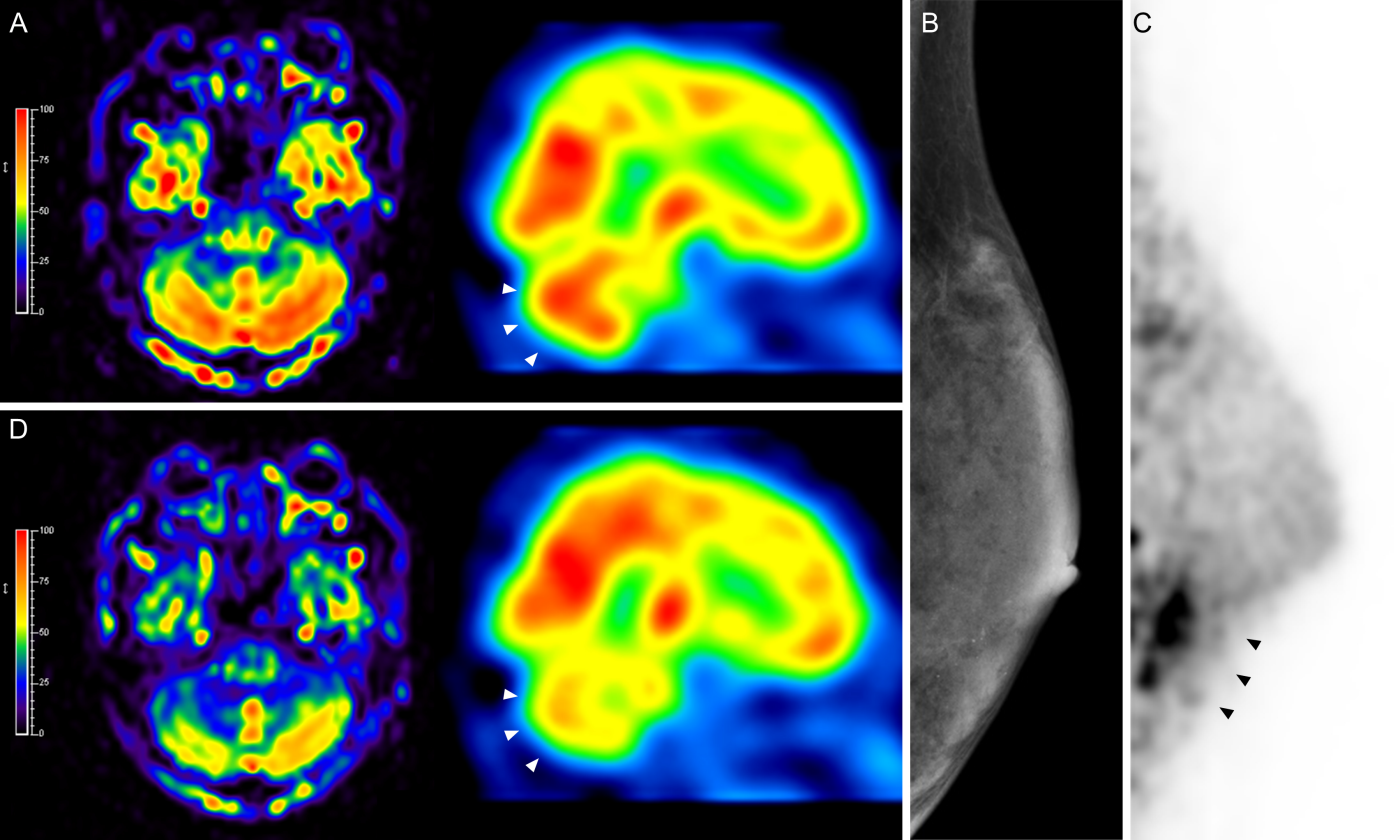
(A) Brain single photon emission computed tomography (SPECT) presented increased cerebellar perfusion (left: coronal; right: sagittal). (B) Mammography did not detect abnormalities. (C) Dedicated breast positron emission tomography presented an abnormal ^18^F-fluorodeoxyglucose uptake in the left breast. (D) Brain SPECT showed a decreased intensity of cerebellar hypermetabolism one week after surgery.

## Article Information

### Author Contributions

All the authors cared for the patient. Shinsuke Sasada drafted the manuscript, and Ayumi Kawamata approved the final version of the manuscript.

### Conflicts of Interest

None

### Approval by Institutional Review Board (IRB)

IRB approval was not required for this study.

### Informed Consent

Informed consent was obtained from the patient.
